# Meconium peritonitis resulting from different etiologies in siblings: a case report

**DOI:** 10.1186/s12887-020-2016-3

**Published:** 2020-03-05

**Authors:** Kyung Hee Park, Mi Hye Bae, Na Rae Lee, Young Mi Han, Shin-Yun Byun, Hae-Young Kim

**Affiliations:** 1 Department of Pediatrics, Pusan National University Hospital, Pusan National University School of Medicine, Busan, South Korea; 2 Department of Pediatrics, Pusan National University Children’s Hospital, Pusan National University School of Medicine, 20 Geumo-ro, Yangsan, 50612 South Korea; 3 Department of Surgery, Pusan National University Hospital, Busan, South Korea

**Keywords:** Meconium peritonitis, Ileostomy, Case report

## Abstract

**Background:**

Meconium peritonitis is defined as aseptic chemical inflammation caused by intrauterine bowel perforation. The underlying causes of bowel perforation include intestinal atresia, midgut volvulus, intussusception, congenital bands, and meconium ileus.

**Case presentation:**

Siblings with prenatally diagnosed meconium peritonitis of different etiologies were found. The elder sister was born at 36 + 6 weeks gestation with a birth weight of 3110 g. She was diagnosed with meconium peritonitis caused by ileal atresia. Two years later, the younger brother was born at 34 + 3 weeks gestation with a birth weight of 2850 g. He was diagnosed with meconium peritonitis caused by midgut volvulus.

**Conclusions:**

Among the previously reported cases of meconium peritonitis, familial occurance of meconium peritonitis is extremely rare. We present a case of prenatally diagnosed meconium peritonitis in siblings to promote further understanding of its etiology and clinical course.

## Background

Meconium peritonitis (MP) is defined as aseptic chemical inflammation caused by an intrauterine bowel perforation. These perforations may result from mesenteric ischemia or obstruction, including intestinal atresia, volvulus, intussusceptions, meconium plug syndrome, inguinal hernia, Hischsprung’s disease, and meconium ileus due to cystic fibrosis, in which the latest is rare in Asian populations [1, 2]. Diagnostic features of MP are abdominal calcifications, ascites, polyhydramnios, meconium pseudocysts, echogenic masses, and a dilated bowel or intestinal obstruction. Recently, an increasing number of diagnoses via prenatal ultrasound have been reported. Clinical results and treatments are dependent on the individual features of MP.

Among the previously reported cases of MP, familial occurance of MP is extremely rare. Only a few cases have been reported. We experienced siblings with prenatally diagnosed MP, caused by ileal atresia and midgut volvulus respectively.

## Case presentation

### Case 1

A 3110 g female baby was born at 36 + 6 weeks gestation by cesarean section. Her mother was a 30-year-old primigravid woman referred to our hospital at 28 weeks of gestation due to fetal ascites and polyhydroamnios, which were suspicious for MP. The patient was the first baby of healthy Korean parents. There was no family history of congenital anomalies. The patient had Apgar scores of 5 and 6 at 1 and 5 min, respectively. The body length was 45 cm (10–25 percentile) and head circumference was 34 cm (75–90 percentile).

**Fig. 1:**
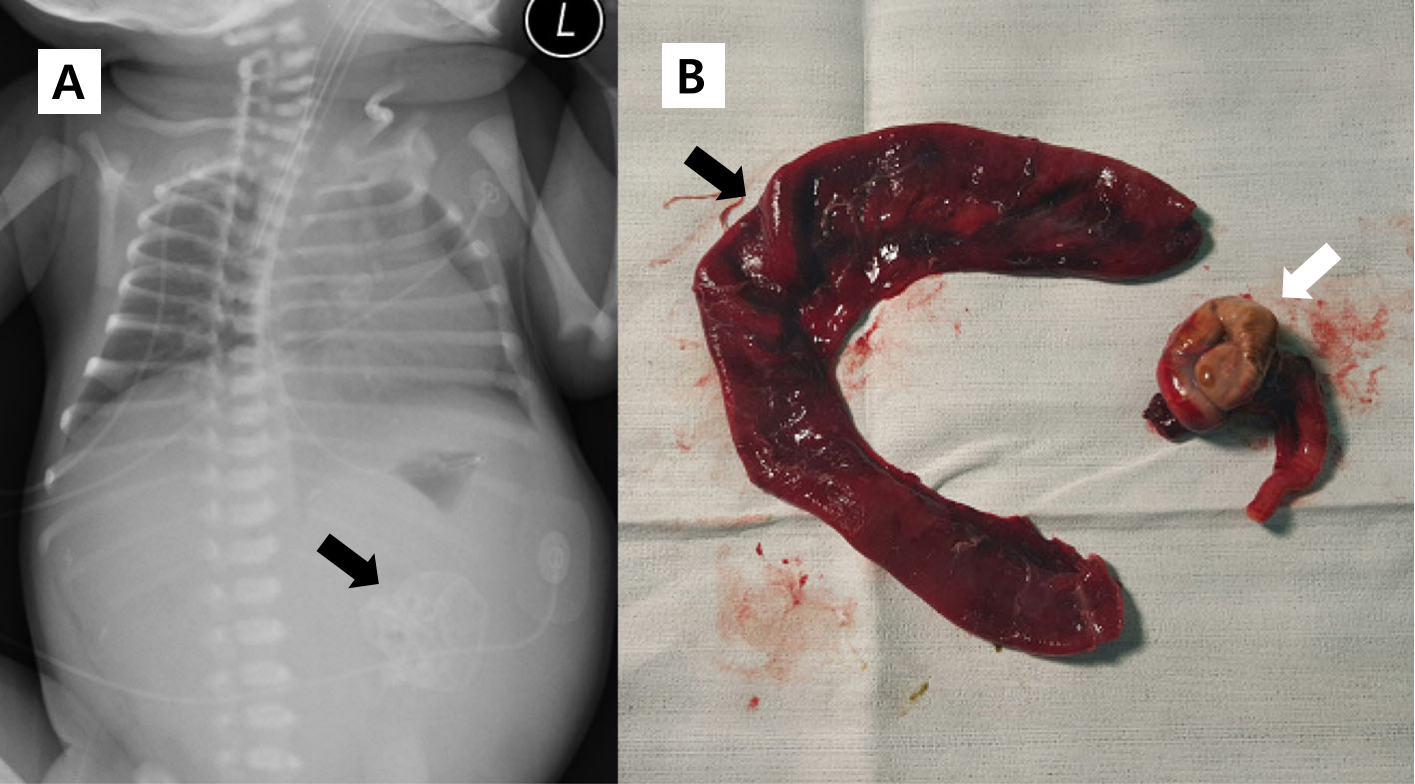
**a** A plain abdominal X-ray showing a calcified mass-like density in the left mid-abdomen (arrow). **b** A markedly distended proximal pouch in the ileum (arrow) and calcified distal pouch (white arrow), was resected

After birth, the baby had respiratory difficulties. She was intubated and mechanically ventilated. She showed mild abdominal distension. A plain abdominal X-ray showed bulging flanks, an elevated diaphragm, and a calcified mass-like density in the left mid-abdomen (Fig. 1a). A laparotomy on the second day of life revealed a large amount of greenish peritoneal fluid with free meconium. A markedly dilated proximal segment ending blindly was found in the ileum 100 cm distal to the Treitz ligament and a perforation was found in the distal pouch corresponding to the calcification in the abdominal X-ray (Fig. 1b). We diagnosed her ileal atresia with perforation in the distal pouch. Affected proximal and distal pouches were resected, and a primary anastomosis was performed.

Her respiratory symptoms improved after operation. She was extubated 1 day post-operatively. Feeding was started on post-operative day 7 and she showed gradual improvement. The remainder of her hospital course was uneventful, and she was discharged at 18 days of life. The patient’s karyotype was normal (46, XX). Laboratory studies were all normal. At her 3-year follow-up, patient condition was good with normal weight gain and development milestones.

### Case 2

The parents had a second pregnancy 2 years later. No fetal abnormalities were detected until 24 weeks of gestation at serial fetal ultrasounds, which revealed a mildly dilated and echogenic bowel and ascites. Serial fetal ultrasound showed increasing ascites. At 34 + 3 weeks gestation, an emergency cesarean section was performed due to fetal distress with prolonged deceleration on a non-stress test.

**Fig. 2:**
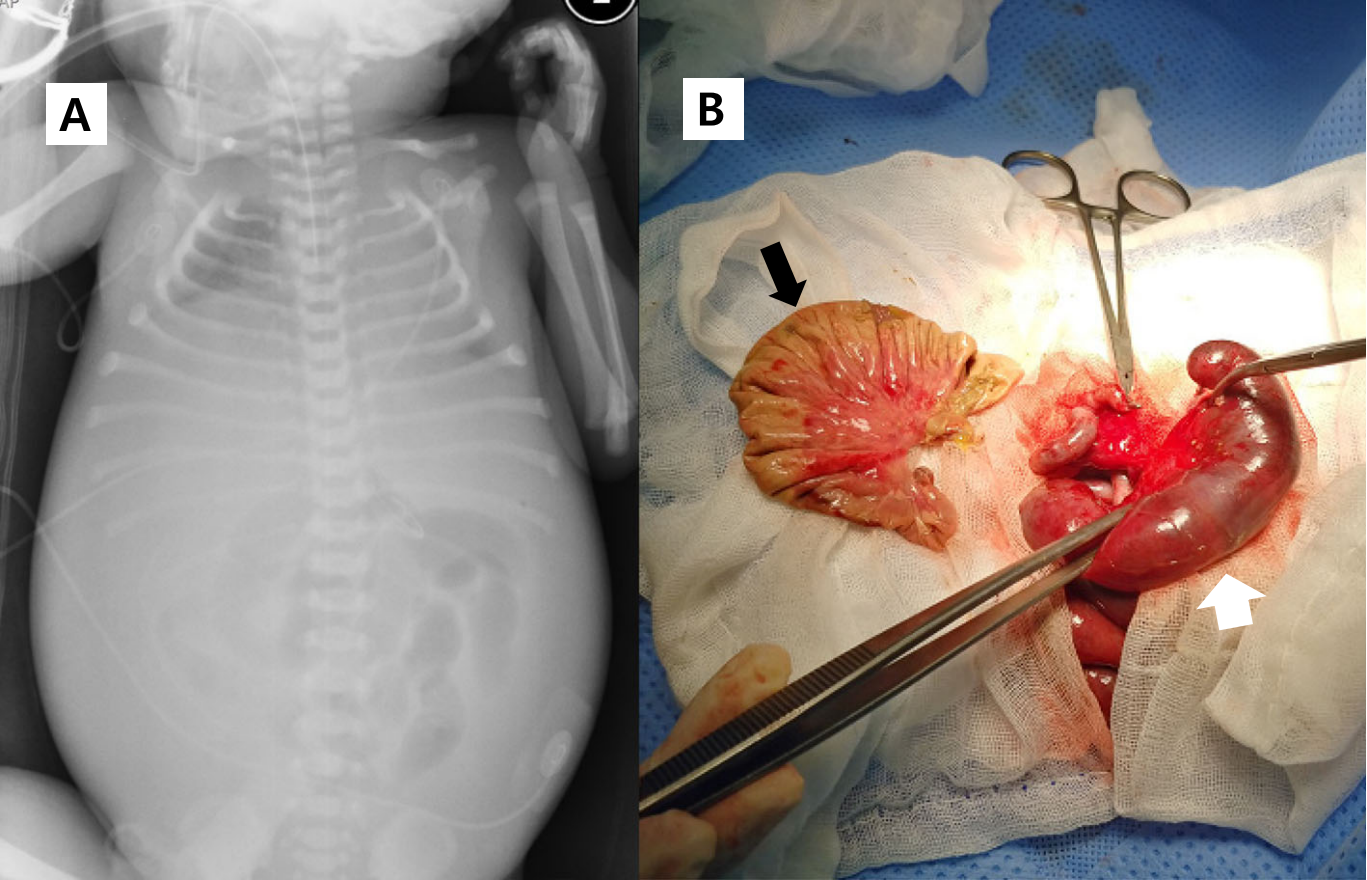
**a** A chest radiograph showing diffuse haziness on both lung fields and a bilaterally elevated diaphragm. **b** The dilated small bowel proximal to volvulus (arrow) was resected and volvus (white arrow) was seen necrotic

The male baby, weighing 2850 g, was delivered with Apgar scores of 4 and 7 at 1 and 5 min, respectively. The baby showed signs of respiratory distress and a markedly distended abdomen. He was intubated and required assisted ventilation. A chest radiograph showed diffuse haziness on both lung fields and a bilaterally elevated diaphragm. An abdominal X-ray showed bulging flanks and a gasless abdomen, but no calcification (Fig. 2a). He was diagnosed with respiratory distress syndrome and surfactant was administrated. Despite surfactant administration, his lungs worsened. Therefore, an emergency laparotomy was performed 3 h after birth. Intraoperative findings showed a 20 cm-sized volvulus in the distal jejunum with meconium-stained ascites occupying the abdomen (approximately 300 ml). A perforation was found in jejunum proximal to volvulus and the affected jejunum was markedly dilated (Fig. 2b). We resected dilated proximal jejunum and distal necrotic intestine of the volvulus and performed a primary anastomosis.

After the operation, his respiratory symptoms improved. However, not long after laparotomy, his lung deteriorated and gradually developed bronchopulmonary dysplasia. Unfortunately, his bowel function did not improve until 2 weeks of life. Therefore, at 15 days of life, an exploratory laparotomy was performed. Severe adhesion was found at around the primary anastomosis site. After peritoneal adhesiolysis, ileostomy 10 cm dital to primary anastomosis site was performed. After the ileostomy, the baby began feeding and tolerated it well. Four weeks later, an ileostomy closure was performed.

He regained bowel function on post-operative day 8 and initiated low volume enteral feeds with gradual advancement. We attempted to wean the patient off mechanical ventilation several times but failed. After steroid administration, he was extubated successfully at 2 months of age but he suffered from oxygen dependency and cholestasis-associated parenteral nutrition during the remainder of his hospitalization. He was discharged at 4 months of age. The patient’s karyotype was normal (46, XY). Laboratory studies, were all normal.

## Discussion and conclusions

Among the previously reported cases of MP, MP in siblings is very rare [3, 4]. So far, only two cases have been reported. First, Shyu et al. reported a patient with MP in Taiwan [3]. That patient had a deceased brother whose autopsy revealed meconium peritonitis and hydrocephalus but no intestinal perforation or malformation. In that patient, intrauterine paracentesis was done and the exact cause of MP remained unknown. We couldn’t know whether that was a case of true familial occurance of MP because one of the siblings was stillborn and neither had any intestinal perforations detected.

Second, Chitayat et al. reported on siblings (a brother and a sister) who presented prenatally with ultrasound findings of MP and were postnatally found to have a perforation of the terminal ileum [4]. However, our patients presented slightly differently from those two previous familial cases. While both of our patients were prenatally diagnosed with MP, the causes of intestinal perforation were different in each sibling (ileal atresia for one sibling, and midgut volvulus for the other).

The pathogenesis of MP is not well understood [4]. Most cases have been idiopathic and sporadic although some cases have been attributed to chromosome abnormalities or cystic fibrosis. Especially, a cystic fibrosis is a known underlying cause of MP in western countries, but is extremely rare in Asian countries [4]. Not only cystic fibrosis is rare in Korean population, but our patients showed intestinal atresia and volvulus rather than meconium ileus [5]. So we could rule out cystic fibrosis.

A decrease in blood flow to the mesentery may lead to mucosal necrosis and subsequent bowel obstruction and perforation of the intestinal wall [6]. According to this theory, it is likely that our patients’ mother was repeatedly exposed to an ischemic environment. However, there was no medical or social history of maternal smoking or vasoconstrictor drug exposure during pregnancy. There is no clear explanation as to why ischemic insults repeatedly occur only in the small intestines of these siblings.

On the other hand, a genetic origin, especially autosomal recessive inheritance, is postulated due to patient reports of affected siblings (different sex) born to unaffected parents. The current case is in accordance with a previous report by Chitayat et al. reporting siblings with MP [4]. However, unfortunately both their patients and our siblings were not performed the genetic test such as whole exome sequencing. In addition, coincidence cannot be ruled out. MP has been reported to have an incidence of one in 30,000–35,000 live births [7–9]. Therefore, the possibility of coincidence like our case must be one in 10^9^.

We do not know whether MP of our case was due to genetic abnormality or coincidence. More studies and case reports including genetic evaluations are required to determine the exact cause of familial occurance of MP including siblings. We present a case of MP in siblings to promote further understanding of the etiology and clinical course. Our case is the first report of MP in siblings resulting from different causes (ileal atresia and midgut volvulus). We need to know that MP was occurred in the siblings, so that we can predict and respond the similar situation in following pregnancy with previous meconium peritonitis history.

## Data Availability

All data generated or analyzed during this study are included in this published article.

## Abbreviation

MP: Meconium peritonitis
